# Use of Video Directly Observed Therapy for Treatment of Latent Tuberculosis Infection — Johnson County, Kansas, 2015

**DOI:** 10.15585/mmwr.mm6614a3

**Published:** 2017-04-14

**Authors:** Elizabeth Lawlor Holzschuh, Stacie Province, Krystle Johnson, Caitlin Walls, Cathy Shemwell, Gary Martin, Amy Showalter, Jennifer Dunlay, Andrew Conyers, Phil Griffin, Nancy Tausz

**Affiliations:** ^1^Johnson County Department of Health and Environment, Olathe, Kansas; ^2^Kansas Department of Health and Environment.

Tuberculosis (TB) is caused by the bacterium *Mycobacterium tuberculosis* and is spread from person to person through the air. TB can be spread in congregate settings, such as school environments, to varying degrees, based on factors including duration of contact and air ventilation (*1*); therefore, evaluating potential contacts and exposures can be challenging. In February 2015, a student at a Kansas high school received a diagnosis of active pulmonary TB disease. Screening of 385 (91%) school contacts, four (100%) household contacts, and 19 (90%) social contacts resulted in the identification of 50 persons with latent TB infection. Johnson County Department of Health and Environment (JCDHE) Public Health Emergency Preparedness personnel used their experience with points of distribution logistics to optimize testing clinic layouts and implement the incident command structure. Open communication with students, school staff members, the public, and the media about the investigation from the outset was imperative to reduce rumors and unease that can accompany a large communicable disease investigation. The large number of persons needing treatment for latent TB overwhelmed JCDHE’s two TB nurses. As a result, JCDHE developed a policy and procedure to allow persons who met eligibility requirements to complete 12 weekly doses of isoniazid and rifapentine treatment using video directly observed therapy (VDOT) rather than traditional in-person directly observed therapy (DOT). This procedure facilitated treatment compliance and completion; among the eligible 15 persons who chose the 12-week VDOT option, 14 (93%) completed treatment. State and local health departments might consider use of VDOT to monitor treatment of persons with latent TB infection.

## Index Patient

On February 27, 2015, JCDHE received notification from an area physician who suspected TB disease in a high school student. The patient had a 3-month history of cough, fatigue, night sweats, 25-pound weight loss, and an abnormal chest x-ray. The patient was immediately placed in home isolation and started on the standard four-drug therapy of isoniazid, rifampin, ethambutol, and pyrazinamide, pending confirmation and susceptibility testing. Sputum specimens were collected from the patient and tested by acid-fast bacilli (AFB) microscopy and culture confirmed at the Kansas Health and Environmental Laboratories. The patient’s sputum was AFB positive, grading 4+, indicating a potentially high level of infectiousness. The specimen was confirmed as TB through nucleic acid amplification testing on March 3, and reported as pansensitive (i.e., sensitive to all antibiotics usually administered in TB treatment) on March 30. Treatment was completed in August 2015.

## Contact Investigation

Contacts of the index patient were identified through interviews with the patient and review of the patient’s class schedule. All four household members tested positive for TB infection by interferon-gamma release assays (IGRAs) and were medically evaluated and determined to have latent TB infection. Twenty-one social contacts were identified, and 19 completed testing, five (26%) of whom had a positive IGRA result and were found to have latent TB infection.

The index patient’s high school has an enrollment of approximately 2,000 students who are predominantly non-Hispanic white (77%) and Hispanic (10%). Initially, JCDHE recommended testing for 345 staff members and students who had at least one class with the index patient. Before the first school clinic, multiple information sessions led by JCDHE TB nurses and the state TB controller were conducted, allowing staff members, students, and parents to ask questions and voice concerns. Joint press releases from JCDHE and the high school were issued. Health department staff members and the school nurse coordinator were available for media interviews.

The first school testing clinic was held on March 11. Local and state health department personnel performed IGRA tests on 282 (81%) students and staff members for whom testing was recommended; all laboratory analyses were performed at the Kansas Health and Environmental Laboratories. After 26 (9%) persons tested positive for likely TB infection, it was learned that nine of the students who tested positive had a weight lifting class with the index patient. Further investigation revealed that 79 students in a second weight lifting class held in the same location had not been identified for the initial testing because the class had a different instructor; this increased the total number of contacts from 345 to 424. The additional 79 students were contacted for testing, information sessions were held, and a second school clinic was conducted on April 8. A third school clinic was conducted on May 5 to ensure that all students and staff members were tested at least 8 weeks after their last exposure to the index patient, to allow sufficient time for seroconversion. During the second and third testing clinics, 15 students were found to have latent TB infection, five of whom were in the second weight lifting class. Overall, among the 424 students and staff members identified for testing, 385 (91%) completed testing, 27 (6%) were tested once but did not complete testing at least 8 weeks following their last exposure to the index patient, and 12 (3%) were never tested. A total of 50 (12%) contacts, including 41 (11%) students and staff members, four household contacts, and five social contacts had positive IGRA results (Table 1).

**TABLE 1:** Testing results and treatment among contacts of a high school student with tuberculosis (TB) disease — Johnson County, Kansas, 2015

Testing results, treatment initiation, and completion	Identified contacts			Total (N = 449)
	Students and staff members (n = 424)	Household contacts (n = 4)	Social contacts (n = 21)	
**No. (%)**	**No. (%)**	**No. (%)**	**No. (%)**
Completed testing*	385 (91)	4 (100)	19 (90)	**408 (91)**
Latent TB infections^†^	41 (11)	4 (100)	5 (26)	**50 (12)**
Initiated treatment**^§^**	41 (100)	4 (100)	5 (100)	**50 (100)**
Completed treatment^¶^	40 (98)	4 (100)	4 (80)	**48 (96)**

* Among identified contacts.

^†^ Among contacts who completed testing.

^§^ Among contacts who tested positive for TB.

^¶^ Among patients who initiated treatment.

## Use of Video Daily Observed Therapy

Medical evaluation ruled out active TB disease in all of the 50 persons who had a positive IGRA result. Therefore, all 50 latent TB patients were offered three treatment options: 1) 9 months of daily isoniazid, self-monitored, with monthly visits to the health department; 2) 4 months of daily rifampin, self-monitored, with visits to the health department every 2 weeks for the first month, and once per month thereafter; or 3) 12 weekly doses of rifapentine and isoniazid administered under DOT.

Sixteen persons selected and completed the 4-month daily rifampin treatment. Seven persons initiated 9-month daily isoniazid treatment, and six completed all 9 months of treatment; one person discontinued treatment for unknown reasons. Twenty-seven of the infected students opted for treatment with the 12 weekly doses of rifapentine and isoniazid under DOT (Table 2).

**TABLE 2:** Treatment regimens and completion rates among latent tuberculosis (TB) infection contacts (N = 50) of a student with tuberculosis (TB) disease — Johnson County, Kansas, 2015

Treatment options for latent TB infection	No. who chose a treatment option	No. (%) who discontinued treatment	No. (%) who completed treatment
Daily isoniazid for 9 months, self-monitored	7	1 (14)	6 (86)
Daily rifampin for 4 months, self-monitored*	16	0 (—)	16 (100)
Weekly rifapentine and isoniazid for 12 weeks, DOT	27	1 (4)	26 (96)
Conventional DOT	12	0 (—)	12 (100)
VDOT	15	1 (7)	14 (93)
**Total**	**50**	**2 (4)**	**48 (96)**

**Abbreviations:** DOT = directly observed therapy; VDOT = video directly observed therapy.

* With visits to the health department every 2 weeks for the first month, and once per month thereafter.

Because the investigation took place in the spring, treatment needed to occur over the summer, making it impossible for JCDHE to partner with the school to manage DOT for students. As a consequence, the number of patients would make it difficult for the two JCDHE TB nurses to provide DOT. Therefore, JCDHE, in consultation with the state TB controller, developed a procedure to implement VDOT. To be eligible for VDOT, patients had to meet specific eligibility requirements (Box).

BOX **Monitoring and eligibility requirements for patients with latent tuberculosis infection choosing video directly observed therapy (VDOT) with a 12-dose regimen of weekly rifapentine and isoniazid — Johnson County, Kansas, 2015**

**Monitoring requirements**

- Obtain baseline laboratory testing before initiation.
- Complete the first 4 doses at the health department, with no complications.
- Have specimens collected at fourth appointment, and receive medication for future VDOT doses.
- Agree to return to the clinic for the eighth and twelfth doses, for clinical evaluation and routine laboratory testing.

**Eligibility requirements**

- No likely risk factors for poor adherence (homelessness, substance abuse, psychiatric illness, reduced mental capacity, or memory impairment).
- Motivated to complete treatment.
- Speak a language that VDOT staff members can accommodate.
- Able to accurately identify medication.
- Access to VDOT device and demonstrate proper use.*
- Physical setting for confidential communication available.
  - All of the eligible patients who participated had their own devices; however, iPads would have been provided if any patients needed them. VDOT was conducted live via FaceTime.

Fifteen of the 27 persons opted for VDOT over conventional DOT. One of the persons being monitored via VDOT discontinued treatment because of an adverse medication reaction.* The remaining 14 persons completed treatment with 100% compliance. Use of VDOT saved JCDHE an estimated $2,066 in mileage and staff time and allowed patients to continue treatment during international travel and family relocation. All 12 students undergoing conventional DOT completed treatment.

## Discussion

The successful investigation and treatment of identified latent TB infection cases can be attributed to extensive collaboration with the school and community. Before laboratory confirmation of TB in the index patient on March 3, 2015, JCDHE developed an incident action plan in partnership with the high school and the state TB controller. JCDHE staff members involved included department leadership, Public Health Emergency Preparedness staff members, public information officers, an epidemiologist, and two TB nurses. The Public Health Emergency Preparedness unit provided expertise in risk communication strategies that were employed throughout the investigation. Communication with the media, high school, and community about the investigation was prioritized, with the first joint press release occurring on March 4. The same day, letters were sent home with all students indicating whether testing was needed. Social media posts and four additional joint press releases informed the public as well as national and international media about the progress of the investigation.

On March 5, state and JCDHE personnel delivered a presentation at the high school to inform students, staff members, and parents about tuberculosis, the investigation process, the importance of being tested, the science behind not testing the entire school, and treatment options if test results were positive. On March 10, an informational forum was held for the public and the media. Throughout the investigation, JCDHE’s TB nurses were available to provide information to concerned school and community members.

The layout of the testing clinic was designed based on points of distribution principles. The incident command structure was implemented during each of the mass testing clinics. After each clinic, volunteers were debriefed and identified successes and opportunities for improvement. This feedback was used to refine the layout and flow of future testing clinics. As of March 2017, no persons had developed TB disease that could be linked to the index patient or the high school.

**Summary**

**What is already known about this topic?**

Tuberculosis (TB) is a contagious airborne disease that can spread in congregate settings such as a school environment. Recommendations for testing contacts in these settings are to test those at highest risk for exposure, followed by evaluation of findings and expanding testing as needed. Persons who test positive for latent TB infection should be treated with an antibiotic course ranging from 12 weeks to 9 months to prevent the development of active TB disease.

**What is added by this report?**

Following identification of a case of infectious TB in a high school student in February 2015, 23 (92%) of 25 household and social contacts and 385 (91%) of 424 high school students and staff members who shared at least one class with the index patient completed TB testing. Among 50 persons who tested positive, all were medically screened, and started on treatment for latent TB infection; 48 (96%) completed treatment. Approximately half (54%) of the infected persons opted for 12 weekly doses of isoniazid and rifapentine treatment, which require directly observed therapy. A procedure was developed to allow these persons to use video directly observed therapy (VDOT) to successfully complete their treatment.

**What are the implications for public health practice?**

VDOT, which previously had only been used during treatment of persons with active TB disease, is a viable option that can reduce costs and the time involved for both TB staff members and patients, while maintaining high compliance and completion rates.
